# Sex-Specific Genetically Predicted Iron Status in relation to 12 Vascular Diseases: A Mendelian Randomization Study in the UK Biobank

**DOI:** 10.1155/2020/6246041

**Published:** 2020-10-26

**Authors:** Fangkun Yang, Qinyi Bao, Zhuo Wang, Menghuai Ma, Jinlian Shen, Feiming Ye, Xiaojie Xie

**Affiliations:** Department of Cardiology, Second Affiliated Hospital, Zhejiang University School of Medicine, Hangzhou, Zhejiang 310009, China

## Abstract

**Background:**

Iron overload has been implicated in the pathogenesis of varicose veins (VVs). However, the association of serum iron status with other vascular diseases (VDs) is not well understood, which might be a potential target for VD prevention. This study was aimed at investigating the causal associations between iron status and VDs using the Mendelian randomization (MR) method.

**Methods:**

A two-sample MR was designed to investigate whether iron status was associated with VDs, based on iron data from a published genome-wide association study meta-analysis of 48,972 subjects of European descent and VD data obtained from the UK Biobank, including 361,194 British subjects (167,020 males and 194,174 females). We further explored whether there was sex difference in the associations between genetically predicted iron status and VDs.

**Results:**

The results demonstrated that iron status had a significant causal effect on VVs of lower extremities (*P* < 0.001) and a potential effect on coronary atherosclerosis (*P* < 0.05 for serum iron, ferritin, and transferrin saturation, respectively), but not on other VDs. Furthermore, higher iron status exerted a detrimental effect on VVs of lower extremities in both genders (*P* < 0.05) and a protective effect on male patients with coronary atherosclerosis (*P* < 0.05 for serum iron, ferritin, and transferrin saturation, respectively).

**Conclusions:**

This MR study provides robust evidence that higher iron status increases the risk of VVs of lower extremities, whereas it reduces the incidence of coronary atherosclerosis in the male population, which indicates that iron has divergent effects on vascular pathology.

## 1. Introduction

Iron is an important trace element that plays critical roles in various biological processes, such as immune function, enzyme activity regulation, and oxygen transport [1], which might contribute to the development of vascular diseases (VDs). Iron deficiency during individual development may have a causal relation with deficits in cognitive or behavioral function [2]. Low serum iron level may have a strong link with venous thromboembolism [3]. Otherwise, iron deficiency has been associated with increased mortality in patients with heart failure. Inconsistently, some studies have demonstrated that iron overload may also have some adverse effects on VDs, such as increasing the propensity for ischemic cardiovascular events by aggravating vascular oxidative stress, increasing risk of thrombosis, and promoting the incidence of type 2 diabetes mellitus [4, 5]. Given the controversy about the role of iron status, it has important clinical significance to investigate the relationship of iron status with VDs, which might be a promising molecular target for VD prevention and therapy.

Observational studies fail to distinguish causal and spurious associations, attributed to the problems of confounding and reverse causality. These shortcomings can be largely overcome by Mendelian randomization (MR) studies. MR is a technique using genetic variants, such as single-nucleotide polymorphisms (SNPs) as proxies for a modifiable exposure (genetic instruments) in order to investigate the causal effect on the risk of the disease [6]. Since genes are allocated randomly at conception, genetic effects on the intermediate phenotype cannot be affected by classical factors, such as lifestyle, or reverse causation, as in the situation where the phenotype level is impacted by the presence of the disease [7]. Moreover, MR analysis can strongly reduce reverse causality bias because genetic variants are always allocated before the onset of the disease [8]. Taken together, MR studies can resemble randomized controlled trials and significantly increase causal inference.

In the present study, we introduced MR design to investigate whether iron status was associated with 12 VDs (including varicose veins of lower extremities, phlebitis and thrombophlebitis, atherosclerosis, coronary atherosclerosis, arterial embolism and thrombosis, pulmonary embolism, peripheral artery disease, varicose veins of other sites, deep vein thrombosis, aortic aneurysm, cerebral aneurysm, and aortic dissection), based on iron data from a published genome-wide association study (GWAS) meta-analysis of 48,972 subjects of European descent and VD data provided by the UK Biobank. Furthermore, we explored whether there was sex difference in the associations between genetically predicted iron status and VDs.

## 2. Materials and Methods

### 2.1. Data Availability

The exposure was the genetically predicted iron status, including serum levels of iron, ferritin, transferrin, and transferrin saturation. Genetic associations with these serum iron biomarkers were obtained from a large-scale meta-analysis of GWAS performed by the Genetics of Iron Status Consortium, adjusted for age and population stratification [9]. The combined analysis of eleven discovery and eight replication cohorts (up to about 50,000 European subjects) identified three SNPs which were significantly associated with all four serum iron biomarkers at a genome-wide threshold of *P* < 5 × 10^−8^. Linkage disequilibrium was tested with LDlink (https://ldlink.nci.nih.gov/) between two loci near the hemochromatosis gene (population: CEU).

The overall and sex-specific summary statistics for the three iron-associated SNPs with 12 VDs were extracted from the UK Biobank [10]. The study included 361,194 British subjects (167,020 males and 194,174 females), adjusted for age and sex. The data was publicly available in Neale lab (http://www.nealelab.is/uk-biobank/) with no restrictions on the use. The UK Biobank and all studies were approved by their institutional review committee, and their participants gave informed consent. Additional sanction for the analysis of summary data was not required.

### 2.2. Data Analyses

The two-sample MR method was applied to evaluate the causal effect of genetically predicted iron status on 12 VDs in the UK Biobank. Increased systemic iron status was defined as the increased serum levels of iron, ferritin, and transferrin saturation and decreased serum levels of transferrin [9, 11]. Three SNPs (rs1800562, rs1799945, and rs855791) were identified independently associated with all four serum iron status markers, as described previously [12, 13]. The effect estimates of each SNP were obtained by using the Wald estimator [14], and the relevant standard error was derived with the delta method [15]. The *F* statistics of the selected SNP was much larger than 10, suggesting the validation of the instrumental variable. Two-sided *P* value < 1.0 × 10^−3^ (=0.05/48, 4 exposures and 12 outcomes) was defined as statistically significant.

MR analyses were performed as follows: (1) overall odds ratio (OR) for each VD was meta-analyzed using the fixed-effect inverse-variance weighted method [16]; (2) sex-specific subgroup analysis of 7 VDs in which summary data was available in the UK Biobank, used the fixed-effect inverse-variance weighted method; (3) sensitivity analysis of the overall results used the simple median and weighted median method, which provided valid estimates if at least 50% of the weight in the analysis came from valid instrumental variables [17]; and (4) pleiotropy analysis used the MR-Egger method, which could detect and adjust for directional pleiotropy [18]. All statistical analyses were performed by using R version 3.6.1 (R Foundation for Statistical Computing, Vienna, Austria) and the MR package.

## 3. Results

The SNP-serum iron status association estimates were obtained from a published GWAS meta-analysis, which comprised 48,972 subjects of European descent from 19 cohorts and was performed by the Genetics of Iron Status Consortium [9]. The data of VDs were obtained from the UK Biobank, including 361,194 British subjects (167,020 males and 194,174 females). There were 14,334 patients with coronary atherosclerosis, 8,763 patients with varicose veins of lower extremities, 6,795 patients with deep vein thrombosis, 2,289 patients with pulmonary embolism, 1,230 patients with peripheral artery disease, 589 patients with aortic aneurysm, 566 patients with atherosclerosis, 510 patients with arterial embolism and thrombosis, 225 patients with cerebral aneurysm, 222 patients with varicose veins of other sites, and 129 patients with aortic dissection (see Table S2).

**Figure 1:**
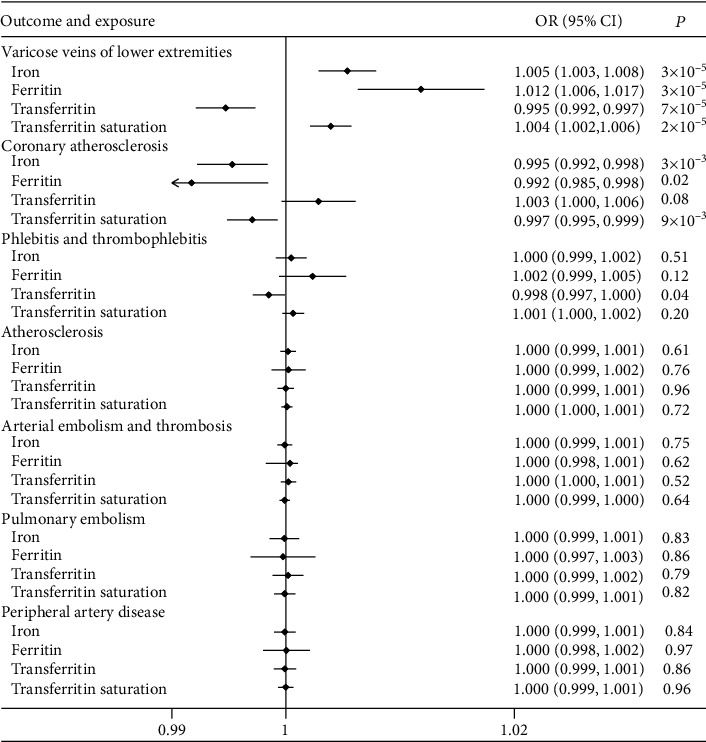
Pooled MR estimates for the effect of iron status on 7 VDs in the UK Biobank. OR: odds ratio; CI: confidence interval; VDs: vascular diseases.

Adjustments were made for age, population stratification (ancestry principal components), and other study specific covariates. The characteristics of SNPs associated with iron status and VDs are shown in Tables S1 and S2, respectively. According to this MR study, genetically predicted iron status had a significant causal effect on VVs of lower extremities and coronary atherosclerosis, but not on other VDs (see Figure 1 and Table S3). It had been demonstrated that there was a detrimental effect of serum iron (OR 1.005; 95% confidence interval (95% CI) 1.003-1.008; *P* = 2.8 × 10^−5^), ferritin (OR 1.012; 95% CI 1.006-1.017; *P* = 2.5 × 10^−5^), and transferrin saturation (OR 1.004; 95% CI 1.002-1.006; *P* = 1.9 × 10^−5^) on the risk of VVs of lower extremities (see Figure 1). In contrast, high serum levels of transferrin (indicated as the lower systemic iron) were associated with a decreased risk of VVs of lower extremities (OR 0.995; 95% CI 0.992-0.995; *P* = 7.3 × 10^−5^; see Figure 1).

As for the arterial diseases, the MR studies showed a potential protective effect of serum iron (OR 0.995; 95% CI 0.992-0.998; *P* = 2.9 × 10^−3^), ferritin (OR 0.992; 95% CI 0.985-0.998; *P* = 0.02), and transferrin saturation (OR 0.997; 95% CI 0.995-0.999; *P* = 9.1 × 10^−3^) on the risk of coronary atherosclerosis (see Figure 1). There was no significant difference in serum transferrin (OR 1.003; 95% CI 1.000-1.006; *P* = 0.08) with the risk of coronary atherosclerosis.

**Figure 2:**
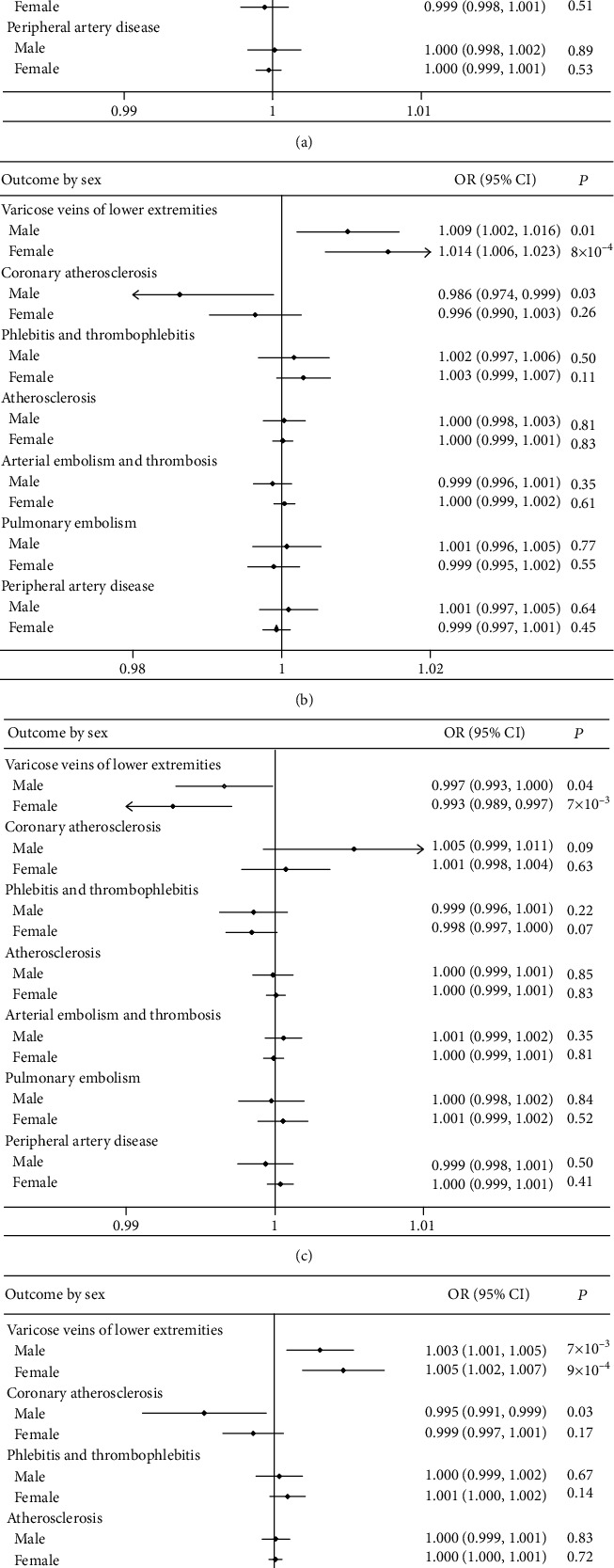
Association of 4 iron biomarkers and 7 VDs in the UK Biobank by sex: (a) iron and vascular diseases, (b) ferritin and vascular diseases, (c) transferrin and vascular diseases, and (d) transferrin saturation and vascular diseases. OR: odds ratio; CI: confidence interval; VDs: vascular diseases.

In addition, there was an obvious sex difference in the associations of genetically predicted iron status with coronary atherosclerosis (Figure 2). The results indicated a protective effect of serum iron (OR 0.993; 95% CI 0.987-0.999; *P* = 0.02), ferritin (OR 0.986; 95% CI 0.974-0.999; *P* = 0.03), and transferrin saturation (OR 0.995; 95% CI 0.991-0.999; *P* = 0.03) in the male population on the risk of coronary atherosclerosis, except for serum transferrin (see Figure 2). There was no significant gender difference in the association of iron status and VVs of lower extremities.

Consistent directional effects for all analyses were observed in the simple median and weighted median sensitivity analyses. The MR-Egger regression method did not provide the evidence of directional pleiotropy in the main results (see Table S4).

## 4. Discussion

Iron is an important trace element involved in fundamental biochemical activities, such as mitochondrial respiration, oxygen delivery, and DNA synthesis in almost all cell types [19]. Iron-mediated injury is noticeably essential in the cardiovascular system [20]. However, it is still controversial whether serum iron status plays a critical role in the development of VDs. The association of serum iron status with a wide range of VDs was systematically investigated in the present study by using the MR approach. Our results firstly showed that higher iron status had a detrimental effect on VVs of lower extremities but a protective effect on coronary atherosclerosis. However, there was no statistically significant association of iron status with other VDs. Furthermore, an obvious sex difference was explored in this study. The protective effect of iron status on the risk of coronary atherosclerosis was only found in the male population. No gender difference was found in the association of iron status and VVs of lower extremities.

### 4.1. Iron and Venous Diseases

Our findings supported the previous observational studies that increasing iron status was associated with the increased risk of VVs of lower extremities [21–24]. No sex difference was found in the association of iron status with VVs of lower extremities. Insufficient evidence indicates that venous vessels are developed by the increasing level of free oxygen radical-induced oxidative DNA damages. Noticeably, the production of free oxygen radicals is intensified by iron overload [23]. T lymphocyte sensitivity might be involved in the increased iron levels in patients suffering from chronic venous diseases such as VVs of lower extremities [25]. Under the conditions of high ferric ion levels, T lymphocytes can differentiate toward the Th1 phenotype, proliferate, and accumulate in the dermis instead of undergoing apoptosis, which might be mediated by the internalization of the R2 chain of the interferon-*γ* receptor, downregulation of nitric oxide synthase expression, and deactivation of caspases [25]. Furthermore, it has been reported that the association of the p.C282Y (rs1800562 A allele, its mutation is regarded as the most common recognized genetic defect in iron metabolism) variant of the hemochromatosis gene contributes to the increased risk of VDs such as venous ulceration and VVs [24, 26]. Taken together, high iron status may lead to the development of VVs of lower extremities.

Previous studies had reported that low serum iron levels were associated with elevated plasma levels of coagulation factor VIII, which might contribute to venous thromboembolism risk [3]. However, the present results showed that there was no statistically significant relation between iron status and pulmonary embolism or deep vein thrombosis. In addition, the present study firstly demonstrated that serum iron may not be involved in the development of phlebitis and thrombophlebitis.

### 4.2. Iron and Arterial Diseases

Consistent with the previous reports [27, 28], our study suggested a protective effect of the increased iron status on the risk of coronary atherosclerosis. Furthermore, it is noticeable that higher iron status has a more protective effect on male patients who suffering from coronary atherosclerosis than on female patients. As reported, iron-induced oxidative stress might be involved in various pathological conditions, including heart failure, cardiomyopathy, and atherosclerosis [29–31]. Cardiovascular mortality was significantly reduced in the patients with stable coronary artery disease accompanied by intrinsic iron release [28], which indicated that iron might be beneficial for coronary artery disease. Inconsistently, other studies reported that iron may aggravate the development of atherosclerosis. It had been demonstrated that iron overload worsens atherosclerosis by increasing the M1 phenotype macrophage population in the wounds of chronic venous leg ulcer in both human and C57BL/6 mice [32]. Proinflammatory M1 phenotype macrophages could express scavenger receptors on their surface and deposit oxidatively modified lipoproteins to atheroma plaques [33]. Moreover, cholesterol accumulation and iron level elevation had been found temporally in human carotid plaque lesions [34]. It had been reported that iron-catalyzed free radical reactions cause oxidation of low-density lipoprotein in the endothelium, smooth muscle cells, lymphocytes, and macrophages, which further triggered the formation of foam cells, the characteristic cells of fatty streak lesions in early atherosclerosis [35, 36]. Furthermore, iron overload might lead to tissue damage and endothelium and smooth muscle cell dysfunction by promoting the generation of reactive oxygen species and finally accelerate the progression of atherosclerosis [20, 37]. Even though lots of animal studies supported that iron administration accelerated atherosclerosis, there has been no evidence to date that increased dietary intake of iron necessarily alters predilection for atherosclerosis [20]. Another study had indicated that higher serum levels of ferritin and transferrin saturation were not associated with an increased extent of coronary atherosclerosis in 100 white subjects who underwent coronary angiography (including 41 females and 59 males) [5]. According to these controversial evidences, further studies are required to verify the roles of iron on vascular pathology.

Although the previous studies suggested that iron could aggravate aortic aneurysm [35], the present MR study indicated that there was no statistical significance between iron status and aneurysm. Tissue level of iron had been found to be higher in the infrarenal aortic wall in patients who underwent surgery for abdominal aortic aneurysm compared to patients with aortic occlusive disease [35]. Otherwise, increased iron levels were also found in serum and aneurismal walls in patients with thoracic aortic aneurysm [38]. However, the results depend on the sites of biopsy specimens, confounding clinical situation and complications, and multiple therapeutic interferences. In contrast to the previous reports that moderate iron loading could accelerate arterial thrombosis [4], the present MR study suggested that there was no significant relation between iron status and arterial thrombosis.

### 4.3. Strengths and Limitations

The strengths and limitations of this MR study merited consideration. Compared with the observational studies, MR analysis can significantly reduce systematic biases such as reverse and confounding causality. In addition, the data used to examine the associations of serum iron status with VVs of lower extremities, coronary atherosclerosis, and other 10 VDs were from large-scale genetic consortia (UK Biobank). Big samples lead to high statistical power. Furthermore, the MR study explored the causal association between iron and VDs. However, some limitations of this study should be acknowledged. Similar to any other MR study, the present study could be affected by selection bias from selecting survivors of their genetic makeup and of competing risk of other specific causes of death that share risk factors [39]. Additionally, the Neale lab only provided the sex-stratified datasets of 7 VDs. Thus, current data were not available to perform sex-stratified analyses for the other 5 VDs. Besides, data cleaning was minimal in the Neale lab GWAS which may lead to inaccurate estimate. Future studies focusing on the relationship between iron status and other VDs would be further accumulated.

## 5. Conclusions

In summary, this MR study supports evidence for a causal association between high serum iron status and the increased risk of VVs of lower extremities in both genders rather than potential decreased risk of coronary atherosclerosis, especially in male subjects. It is indicated that iron plays a divergent role in vascular pathology.

## Data Availability

The data used to support the findings of this study are available from the corresponding author.

## Supplementary Materials

Supplementary Materials Supplementary Table S1: characteristics of SNPs associated with iron status. Supplementary Table S2: general information of vascular disease outcomes in the UK Biobank. Supplementary Table S3: association of iron biomarkers with other 5 vascular disease outcomes in the UK Biobank. Supplementary Table S4: Association of genetically predicted iron status with vascular disease outcomes in sensitivity analyses.
