# Supplementary material for: Sex-Specific Genetically Predicted Iron Status in relation to 12 Vascular Diseases: A Mendelian Randomization Study in the UK Biobank
Source: Biomed Res Int. 2020 Oct 26;2020:6246041. doi: 10.1155/2020/6246041 (PMC7641690; doi:10.1155/2020/6246041)
Supplement: Supplementary Materials — Supplementary Table S1: characteristics of SNPs associated with iron status. Supplementary Table S2: general information of vascular disease outcomes in the UK Biobank. Supplementary Table S3: association of iron biomarkers with other 5 vascular disease outcomes in the UK Biobank. Supplementary Table S4: Association of genetically predicted iron status with vascular disease outcomes in sensitivity analyses. [file 6246041.f1.pdf]

Table S1. Characteristics of SNPs associated with iron status.

CHR	SNP	BP	Nearest Gene	EA	OA	EAF	Phenotype	Beta	SE	*p* value
6	rs1800562	26093141	*HFE(C282Y)*	A	G	0.067	Iron	0.328	0.016	2.72E-97
							Ferritin(log)	0.204	0.016	1.54E-38
							Transferrin	-0.479	0.016	8.90E-196
							Saturation	0.577	0.016	2.19E-270
6	rs1799945	26091179	*HFE(H63D)*	C	G	0.850	Iron	-0.189	0.010	1.10E-81
							Ferritin(log)	-0.065	0.010	1.71E-10
							Transferrin	0.114	0.010	9.36E-30
							Saturation	-0.231	0.010	5.13E-109
22	rs855791	37462936	*TMPRSS6(V736A)*	A	G	0.446	Iron	-0.181	0.007	1.32E-139
							Ferritin(log)	-0.055	0.007	1.38E-14
							Transferrin	0.044	0.007	1.98E-09
							Saturation	-0.190	0.008	6.41E-137

CHR, chromosome; SNP, single-nucleotide polymorphism; EA, Effect allele; OA, Other allele; EAF, frequency of effect allele.

Table S2. General information of vascular disease outcomes in UK Biobank.

Outcome	Number of cases	Number of controls	Number of individuals	Source
Coronary atherosclerosis	14334	346860	361194	finngen
Varicose veins of lower extremities	8763	352431	361194	icd10
Deep vein thrombosis	6795	354399	361194	phesant
Phlebitis and thrombophlebitis	2289	358905	361194	icd10
Pulmonary embolism	2118	359076	361194	icd10
Peripheral artery disease	1230	359964	361194	finngen
Aortic aneurysm	589	360605	361194	finngen
Atherosclerosis	566	360628	361194	icd10
Arterial embolism and thrombosis	510	360684	361194	icd10
Cerebral aneurysm	225	360969	361194	finngen
Varicose veins of other sites	222	360972	361194	icd10
Dissection of aorta	129	361065	361194	finngen

icd10, generated from UK Biobank fields 41202-0.0 - 41202-0.379.
finngen, manually curated by collaborators in the FinnGen research project.
phesant, automatically processed using a modified version of the software PHESANT (https://www.ncbi.nlm.nih.gov/pubmed/29040602).

Table S3. Association of iron biomarkers with other 5 vascular disease outcomes in UK Biobank.

Outcome and Exposure	Iron		Ferritin		Transferrin		Saturation	
	OR(95%CI)	*p* value	OR(95%CI)	*p* value	OR(95%CI)	*p* value	OR(95%CI)	*p* value
Varicose veins of other sites	1.000(0.999,1.000)	0.68	1.000(0.999,1.001)	0.76	1.000(0.999,1.000)	0.44	1.000(1.000,1.000)	0.95
Deep vein thrombosis	0.943(0.851,1.045)	0.26	0.866(0.694,1.080)	0.20	1.067(0.961,1.185)	0.23	0.956(0.889,1.028)	0.22
Aortic aneurysm	1.000(1.000,1.001)	0.14	1.001(1.000,1.002)	0.15	1.000(0.999,1.000)	0.23	1.000(1.000,1.001)	0.15
Cerebral aneurysm	1.000(1.000,1.000)	0.79	1.000(0.999,1.001)	0.62	1.000(1.000,1.001)	0.44	1.000(1.000,1.000)	0.64
Dissection of aorta	1.000(1.000,1.000)	0.85	1.000(0.999,1.001)	0.78	1.000(1.000,1.000)	0.75	1.000(1.000,1.000)	0.80

OR, odds ratio; CI, confidence interval.

Table S4. Association of genetically predicted iron status with vascular disease outcomes in sensitivity analyses.

		Iron		Ferritin		Transferrin		Saturation	
	**Method**	**OR(95% CI)**	**P-value**	**OR(95% CI)**	**P-value**	**OR(95% CI)**	**P-value**	**OR(95% CI)**	***p* value**
Varicose veins of lower extremities	Simple median	1.006(1.003,1.010)	1.4E-04	1.011(1.003,1.019)	0.01	0.988(0.980,0.996)	4.3E-03	1.004(1.001,1.006)	1.5E-03
	Weighted median	1.006(1.003,1.009)	2.0E-04	1.011(1.004,1.017)	7.3E-04	0.995(0.992,0.997)	1.2E-04	1.004(1.002,1.006)	3.4E-04
	MR-Egger	1.009(0.998,1.020)	0.10	1.009(0.999,1.020)	0.09	0.997(0.993,1.000)	0.06	1.004(1.000,1.007)	0.07
Varicose veins of other sites	Simple median	1.000(0.999,1.000)	0.36	0.999(0.997,1.001)	0.33	1.001(1.000,1.003)	0.13	1.000(0.999,1.000)	0.33
	Weighted median	1.000(0.999,1.000)	0.68	1.000(0.999,1.001)	0.64	1.000(0.999,1.000)	0.47	1.000(1.000,1.000)	0.87
	MR-Egger	1.002(1.000,1.003)	0.06	1.002(1.000,1.003)	0.07	0.999(0.999,1.000)	0.16	1.001(1.000,1.001)	0.11
Phlebitis and thrombophlebitis	Simple median	0.999(0.997,1.001)	0.40	0.997(0.991,1.003)	0.37	1.003(0.998,1.007)	0.26	0.999(0.997,1.001)	0.36
	Weighted median	1.000(0.998,1.002)	0.94	1.002(0.999,1.005)	0.18	0.998(0.997,1.000)	0.04	1.001(0.999,1.002)	0.36
	MR-Egger	1.008(1.003,1.014)	3.0E-03	1.008(1.003,1.013)	3.1E-03	0.997(0.995,0.999)	0.01	1.003(1.001,1.005)	3.5E-03
Deep vein thrombosis	Simple median	0.925(0.814,1.052)	0.23	0.839(0.621,1.133)	0.25	1.078(0.893,1.301)	0.44	0.940(0.855,1.033)	0.20
	Weighted median	0.918(0.814,1.035)	0.16	0.835(0.657,1.061)	0.14	1.065(0.956,1.187)	0.25	0.939(0.867,1.018)	0.12
	MR-Egger	0.818(0.523,1.278)	0.38	0.822(0.525,1.287)	0.39	1.061(0.894,1.258)	0.50	0.931(0.777,1.115)	0.44
Atherosclerosis	Simple median	1.000(0.999,1.001)	0.91	1.000(0.998,1.002)	0.94	1.000(0.999,1.001)	0.96	1.000(0.999,1.001)	0.93
	Weighted median	1.000(0.999,1.001)	0.84	1.000(0.998,1.002)	0.98	1.000(0.999,1.001)	0.92	1.000(0.999,1.001)	0.99
	MR-Egger	0.999(0.997,1.002)	0.68	0.999(0.997,1.002)	0.67	1.000(0.999,1.001)	0.62	1.000(0.999,1.001)	0.65
Coronary atherosclerosis	Simple median	0.994(0.990,0.999)	0.01	0.984(0.970,0.998)	0.02	1.009(0.999,1.019)	0.07	0.995(0.992,0.999)	0.02
	Weighted median	0.994(0.991,0.998)	3.1E-03	0.992(0.985,0.999)	0.03	1.002(0.999,1.006)	0.15	0.997(0.995,1.000)	0.02
	MR-Egger	1.003(0.991,1.016)	0.63	1.003(0.991,1.015)	0.63	0.999(0.995,1.003)	0.62	1.001(0.996,1.006)	0.62
Arterial embolism and thrombosis	Simple median	1.000(0.999,1.001)	0.49	0.999(0.998,1.001)	0.58	1.000(0.999,1.002)	0.72	1.000(0.999,1.000)	0.52
	Weighted median	1.000(0.999,1.000)	0.47	1.000(0.998,1.001)	0.54	1.000(1.000,1.001)	0.48	1.000(0.999,1.000)	0.51
	MR-Egger	0.999(0.997,1.002)	0.51	0.999(0.997,1.002)	0.51	1.000(0.999,1.001)	0.47	1.000(0.999,1.001)	0.49
Pulmonary embolism	Simple median	1.000(0.998,1.002)	0.94	1.000(0.996,1.004)	0.96	1.000(0.997,1.003)	0.98	1.000(0.999,1.001)	0.95
	Weighted median	1.000(0.999,1.002)	0.79	1.000(0.997,1.003)	0.89	1.000(0.999,1.002)	0.80	1.000(0.999,1.001)	0.89
	MR-Egger	1.000(0.992,1.008)	0.97	1.000(0.992,1.008)	0.98	1.000(0.997,1.003)	0.93	1.000(0.997,1.003)	0.98
Aortic aneurysm	Simple median	1.001(1.000,1.001)	0.15	1.001(0.999,1.003)	0.34	1.000(0.998,1.001)	0.52	1.000(1.000,1.001)	0.27
	Weighted median	1.001(1.000,1.001)	0.11	1.001(0.999,1.003)	0.21	1.000(0.999,1.000)	0.31	1.000(1.000,1.001)	0.19
	MR-Egger	1.001(0.998,1.003)	0.63	1.001(0.998,1.003)	0.64	1.000(0.999,1.001)	0.71	1.000(0.999,1.001)	0.67
Cerebral aneurysm	Simple median	1.000(0.999,1.000)	0.45	1.000(0.999,1.001)	0.55	1.000(0.999,1.001)	0.66	1.000(1.000,1.000)	0.51
	Weighted median	1.000(0.999,1.000)	0.74	1.000(0.999,1.001)	0.61	1.000(1.000,1.001)	0.39	1.000(1.000,1.000)	0.59
	MR-Egger	0.999(0.997,1.002)	0.63	0.999(0.997,1.002)	0.62	1.000(0.999,1.001)	0.51	1.000(0.999,1.001)	0.57
Aortic dissection	Simple median	1.000(1.000,1.000)	0.99	1.000(0.999,1.001)	0.99	1.000(0.999,1.001)	0.99	1.000(1.000,1.000)	0.99
	Weighted median	1.000(1.000,1.000)	0.99	1.000(0.999,1.001)	0.80	1.000(1.000,1.000)	0.75	1.000(1.000,1.000)	0.83
	MR-Egger	1.000(0.999,1.001)	0.74	1.000(0.999,1.001)	0.74	1.000(1.000,1.000)	0.74	1.000(0.999,1.000)	0.74
Peripheral artery disease	Simple median	1.000(0.999,1.001)	0.81	1.000(0.996,1.003)	0.76	1.001(0.998,1.003)	0.61	1.000(0.999,1.001)	0.77
	Weighted median	1.000(0.999,1.001)	0.94	1.000(0.998,1.002)	0.84	1.000(0.999,1.001)	0.78	1.000(0.999,1.001)	0.91
	MR-Egger	1.001(0.997,1.005)	0.52	1.001(0.997,1.005)	0.53	1.000(0.998,1.001)	0.58	1.000(0.999,1.002)	0.55

OR, odds ratio; CI, confidence interval.
